# Risk genes in progressive supranuclear palsy (PSP) affect integrity and function of microtubules

**DOI:** 10.3389/fragi.2026.1769377

**Published:** 2026-04-20

**Authors:** Timothy A. Donlon, Ulrich Müller

**Affiliations:** 1 Department of Research, Kuakini Medical Center, Honolulu, HI, United States; 2 Department of Cell and Molecular Biology, John A. Burns School of Medicine, University of Hawai’i at Mānoa, Honolulu, HI, United States; 3 Institute of Human Genetics, Justus-Liebig-University, Giessen, Germany; 4 Center for Human Genetics, MVZ Diagnosticum, Frankfurt, Germany

**Keywords:** autophagosome, dementia, microtubule, progressive supranuclear palsy, unfolded protein

## Abstract

Progressive supranuclear palsy (PSP) is a tauopathy and has a multifactorial etiology. The genetic component comprises at least 15 genes with unrelated functions that increase risk for PSP with a high degree of certainty. The function of these genes in increasing risk for PSP is presently unknown. This study was undertaken to identify new pathological pathways of these genes/proteins in increasing risk for PSP. Identification of possible targets and pathways of these genes was investigated using publicly available databases. 13 out of 15 of the risk genes, i.e. *MAPT, KANSL1, PLEKHM1, STX6, MOBP, EIF2AK3, DUSP10, APOE, RUNX2, TRIM11, NFASC, CNTN2*, and *LRRK2* target microtubules, and directly alter their function via variable mechanisms. We now present data that these pathways are predicted to involve common pathways strongly involving microtubule hemeostasis, such as vesicle transport of misfolded proteins to lysosomes and cellular export. Two genes (*SLCO1A2* and *C4A*) are not obviously directly targeting microtubules. Mutations of the risk genes interfere with microtubular function and/or structure as they relate to axon formation/integrity, axon transport, intracellular organelle transport and communication, and cellular, microtubule - guided waste management. Microtubules may be thought of as a conveyor belt for the distribution of nutrients and waste management. Taken together these alterations include an increased risk of tau precipitation (MAPT) and are molecular drivers of neuronal degeneration in PSP. Although microtubular dysfunction has long been documented in PSP mainly based on the findings related to MAPT, this is the first study of the effect of risk genes in PSP. We demonstrate that most of these genes (13/15) also affect microtubular structure and function. These genes/proteins may also be biased towards neurodegeneration in motor neurons.

## 1 Introduction

Progressive supranuclear palsy (PSP) is a tauopathy. Age of onset of PSP most commonly lies between the mid- and late sixties. The typical clinical sign of PSP is vertical supranuclear gaze palsy accompanied by postural instability with difficulty walking and frequent falls, axial rigidity, dysarthria, dysphagia, and frequently frontal lobe cognitive disturbances and dementia. PSP is a disorder with multifactorial etiology and thus mainly occurs sporadically (Höglinger et al., 2017).

Neurodegeneration occurs in various regions of the brain including the motor cortex, basal ganglia, tegmentum, base of the pons, cerebellum, and medulla. Cytoplasmic aggregates are formed by impeded binding of abnormally posttranslational modified tau to microtubules causing their destabilization. This in turn disturbs cellular structure and functions. Cytoskeletal breakdown results in various lesions such as tufted astrocytes, globose neurofibrillary tangles in neurons and coiled bodies in oligodendrocytes (Dickson et al., 2007).

A recent article of GWAS in PSP (Müller et al., 2025) reviewed the genetic component of this multifactorial disorder and identified 14 highly probable (significance *P* < 5^−8^) risk genes: *MAPT, KANSL1, PLEKHM1, STX6, MOBP, EIF2AK3, SLCO1A2, DUSP10, APOE, RUNX2, TRIM11, NFASC/CNTN2 and LRRK2.* An additional potential risk gene, *C4A,* was identified in a gene dense region of 6p21.32. Transcriptomic, histological and biochemical analyses suggest that *C4A* is a risk gene in PSP as well. Here we describe the physiological function of the 15 risk genes, 13 of which directly affect structure and function of microtubules. Nine (*MAPT, CNTN2/NFASC, MOBP, EIF2AK2, APOE, KANSL1, RUNX2, DUSP10*) interfere with microtubule homeostasis, and four (*LRRK1, PLEKHM1, STX6, TRIM1*) affect microtubular stability and transport and/or cytoskeletal homeostasis and organelle/vesicle transport (summarized in Table 1).

**TABLE 1:** Genes associated with PSP and their roles in microtubule homeostasis.

Gene/Protein	Cellular function	Mechanism of action	References
*MAPT*	**Microtubule stabilization, axon transport, and vesicle transport**	Binds to and stabilizes **microtubules** in neurons, oligodendrocytes, and astrocytes. Abnormal or dysregulated tau does not properly bind to microtubules thus interfering with their homeostasis. This leads to disturbed axonal transport in neurons and degeneration of the nervous system	Guo et al. (2017)
*LRRK2*	**Microtubule stabilization, axon transport**	**Stabilizes the cytoskeleton**, blocks the function of the motor molecules dynein and kinesin thus regulating intracellular trafficking and axonal transport	Parisiadou and Cai (2010), Pellegrini et al. (2017), Snead et al. (2022)
*APOE*	Lipid metabolism, **microtubule stabilization**	APO ε4 allele enhances tau spread and inflammation in tauopathies	Cervós-Navarro and Schumacher (1994), Strittmatter et al. (1994), Scott et al. (1998), Nathan et al. (1995)
*CNTN2/NFASC*	**Microtubule stabilization, axon transport**	Functions are important for synapse formation and preservation of structure and function of neurons Stabilize synaptic contacts and interact with and **stabilize microtubules** in neurons	Masuda (2017)
*KANSL1*	**Microtubule stabilization**	Abnormal KANSL1 disturbs cellular integrity. Deficiency results in developmental defects associated with the Koolen-de Vries syndrome	Pavlova et al. (2019)
*DUSP10*	MAPK pathway regulation, **microtubule stabilization**	Regulates the p38 MAPK pathway, which can influence **microtubule dynamics and stability**	Li et al. (2015), Jiménez-Martínez et al. (2019)
*RUNX2*	**Microtubule stabilization** and autophagy	**Stabilizes microtubules** and facilitates autophagy and contributes to cell survival. Microtubules are also involved in nuclear-cytoplasmic shuttling of RUNX.	Othman et al. (2022)
*PLEKHM1*	Endolysosomal **vesicle transport**, autophagy regulation	Late stages of endolysosomal maturation, facilitating both endocytosis-mediated degradation of growth factor receptors and **autophagosome clearance** and promotes cargo traffic to lysosomes	Tabata et al. (2010), Marwaha et al. (2017)
*STX6*	Golgi and endosomal **vesicle trafficking**	Directs movement of vesicles from endosomes to the cell membrane. Interacts with tau and enables **vesicle release** from cells via secretory pathways	Jones et al. (2024)
*MOBP*	Myelin structural protein, **vesicle transport**	Interacts with the microtubular network in oligodendrocytes, affecting **microtubule organization, stability, and transport**	Torii et al. (2022)
*TRIM11*	ER stress/UPR pathway, **vesicle transport**	Removes the cellular **misfolded proteins** or protein aggregates via the unfolded protein system (UPS) pathway. By mediating degradation of both mutant and normal tau it protects against tauopathies	Zhang et al. (2023)
*EIF2AK3*	ER stress/UPR pathway, **vesicle transport**	Protects against tau aggregation suggesting a role in maintaining microtubule-associated protein stability under stress conditions involving the **unfolded protein response**	Sánchez-Álvarez et al. (2025), Smedley et al. (2021)
*SLCO1A2*	Organic anion transporter	May affect drug/xenobiotic transport at the blood-brain barrier; mechanistic role in PSP is unclear	Uhlén et al. (2015)
*C4A*	Complement system	Contributes to Multiple Sclerosis pathology and disease progression	Farrell et al. (2024), Gracias et al. (2022), Westacott and Wilkinson (2022)

The gene symbol is shown in the first column, followed by the cellular function, the mechanisms of action, and appropriate references. Microtubule-related pathways and vesicle transport pathways are denoted in bold.

Although a role of microtubular dysfunction in PSP has been shown, mainly based on MAPT, the present investigation studies the effects of all highly probable risk genes in PSP for their effects on microtubules. The genes/proteins included in this study were taken from the previous publication by Müller et al. (2025). Possible roles in microtubule homeostasis for these genes/proteins were searched, using PubMed, to include the following terms: microtubules, homeostasis, tubulin, misfolded protein, unfolded protein, ER stress, palsy, and progressive supranuclear palsy. Gene/protein functional information was also deduced from GeneCards (Safran et al., 2022; Stelzer et al., 2016), GeneCaRNA (Barshir et al., 2021), and On-line Mendelian Inheritance in Man (https://www.ncbi.nlm.nih.gov/omim?db=OMIM).

## 2 Microtubule homeostasis

Microtubules provide polarized tracks for directional transport of nutrients and various cellular organelles. Each microtubule has a + end (dynamic, toward axon terminal) and a – end (more stable, often toward the soma). This polarity is essential because molecular motors read it like a map: Kinesin motors move toward the + end for an anterograde transport (soma → axon terminal). Dynein motors move toward the – end for a retrograde transport (terminal → soma). Different cargos must go to specific neuronal compartments and include: 1) delivery of synaptic vesicle precursors to the synaptic terminals, 2) delivers endosomes to autophagosomes and/or the lysosomes for processing and/or degradation, 3) delivery of mitochondria to regions of high metabolic demand. This long-distance organization is not possible with actin-based transport alone and requires microtubules. We report that nine (*MAPT, CNTN2/NFASC, MOBP, EIF2AK2, APOE, KANSL1, RUNX2, and DUSP10*) of the 15 risk genes/proteins affect microtubule stability and/or homeostasis. These structures include axon transport of micronutrients, providing addresses for the delivery of cellular cargo, and the transport of cellular organelles. Posttranslational modifications of microtubules determine cargo specificity. These modifications primarily include: 1) acetylation, which increases kinesin-1 transport efficiency, 2) detyrosination, which stabilizes microtubules, promotes dynein processivity, 3) polyglutamylation, which regulates binding of severing enzymes and motors, and 4) phosphorylation, glycylation, which involves compartment-specific tuning. These PTMs allow the cell to route different organelles down different microtubule subsets.

## 3 Axon stability and transport

*MAPT* (Microtubule-Associated Protein Tau) encodes protein tau that binds to and stabilizes microtubules in neurons, oligodendrocytes, and astrocytes. Hyperphosphorylation of tau can lead to disturbed axonal transport, microtubular disassembly, and ultimately to tau protein aggregation and cell death (Guo et al., 2017).

*LRRK2* encodes Leucine Rich Repeat Kinase 2. Mutations of this gene are a common cause of familial Parkinson disease (PD). LRRK2 interacts with microtubules to modify microtubule dynamics (Parisiadou and Cai, 2010). It stabilizes the cytoskeleton, blocks the function of the motor molecules dynein and kinesin thus regulating intracellular trafficking and axonal transport. LRRK2 interactions with the cellular microtubule network are disturbed when LRRK2 is mutated as in familial PD. This lack of functional interaction results in neurodegeneration (Snead et al., 2022). Interaction of LRRK2 with microtubules is also a prerequisite for neuronal outgrowth (Pellegrini et al., 2017).

*APOE* affects microtubules through lipid trafficking and signaling. The APOE protein (Apolipoprotein E) is highly expressed in the brain, particularly in the basal ganglia, midbrain and pons and somewhat less in other brain structures. There are three major isoforms, referred to as ApoE2, ApoE3 and ApoE4. ApoE4 increases and ApoE2 decreases the risk for Alzheimer disease (AD). The opposite applies to PSP (Cervós-Navarro and Schumacher, 1994). ApoE binds to microtubules. This was shown in neurofibrillary tangles (NFT) found in the brains of patients with AD and PSP. NFT found in AD are formed from hyperphosphorylated tau associated with microtubules. Specifically, ApoE3 binds to the microtubule - binding repeat region of tau. In PSP NFT appear to differ from those found in AD. While paired helical filaments are typical for AD, NFT in PSP are composed of straight filaments and microtubules. These observations indicate different types of cytoskeletal disorganization in AD and PSP (Strittmatter et al., 1994). Further evidence for an effect of ApoE on microtubules comes from *in vitro* experiments. Thus Scott et al. (1998) showed an ApoE-mediated acceleration of microtubule polymerization and the experiments of Nathan et al. (1995) demonstrated that ApoE4 affects neuronal growth by de-polymerizing microtubules. In addition, APOE isoforms (especially APOE4) remodel endosomal–lysosomal trafficking: enlarged Rab5/Rab7 endosomes, altered recycling, and changed Aβ routing through Rab5/7-positive compartments in neurons and glia (Nuriel et al., 2017).

*CNTN2/NFASC* encode neurofascin and contactin-2, respectively. The genes are about 20 kb apart and have partially complementary roles in the nervous system. Neurofascin is involved in myelination, formation of paranodal junctions at the Ranvier nodes, and conduction of nerve impulses; contactin-2 is involved in myelination as well, and is required for formation of paranodal junctions at the Ranvier nodes and maintains voltage-gated potassium channels (Hivert et al., 2016). Although not cytoskeletal proteins *per se*, CNTN2 and NFASC anchor ankyrin–spectrin scaffolds at the axon initial segment and nodes of Ranvier (Kalafatakis et al., 2021). These scaffolds form microtubule-anchoring and cargo-filtering zones that maintain axonal polarity. Disruption alters the organization of microtubule arrays and the directed movement of vesicles and ion-channel–containing transport packets. The functions of both molecules are important for synapse formation and preservation of structure and function of neurons. Both molecules stabilize synaptic contacts and interact with microtubules in neurons. These interactions are essential for maintenance of the neuronal cytoskeleton. This ensures function of synapses, axons, axonal transportation and additional roles of neurofascin and contactin-2 (Masuda, 2017; Parato and Bartolini, 2021). The two genes have been treated in one paragraph due to their close proximity and similar functions.

*KANSL1* (KAT8 Regulatory NSL Complex Subunit 1) KANSL1 is part of the histone acetyltransferase (HAT) complex NSL that functions as a microtubule minus-end (i.e., the slower growing end of microtubules) binding and stabilizing factor during mitosis. By interacting with KANSL3 and MCRS1 it sustains chromosomal microtubules and kinetochore fibers, ensuring proper chromosome segregation in mitosis (Pavlova et al., 2019). Abnormal KANSL1 thus disturbs cellular integrity.

*DUSP10* encodes the Dual Specificity Protein Phosphatase 10 - also known as MKP5 (Map Kinase Phosphatase 5) - regulates the p38 MAPK pathway which can influence microtubule dynamics (Li et al., 2015). DUSP10 selectively dephosphorylates stress-activated kinases with specificity to the p38 and JNK/SAPK kinases. Thus, DUSP10 plays a role in microtubule malfunction (Jiménez-Martínez et al., 2019).

*RUNX2* influences microtubules primarily through transcriptional regulation: Regulates cytoskeletal-associated genes: it controls expression of MAPs, tubulin-modifying enzymes, and motor protein regulators and is a ubiquitous transcription factor. In neuronal tissues it regulates development and growth of neurons and modulates neuronal regeneration (Wang and Stifani, 2017). RUNX2 stabilizes microtubules by inhibiting HDAC6 (Histone Deacetylase 6) interaction with α-tubulin, which results in an increase of acetylation. This stabilization facilitates autophagy and contributes to cell survival (Othman et al., 2022). Microtubules are also involved in nuclear-cytoplasmic shuttling of RUNX2 (Pockwinse et al., 2006).

## 4 Cytoskeleton homeostasis and organelle/vesicle transport

Microtubules play a major role in vesicular trafficking processes (Qu et al., 2025). Microtubules organize cellular organelles in their respective compartments, however, microtubule-based motors do more than move organelles as they keep them strategically localized. Without them organelles become mispositioned, fragmented, and functionally impaired. Many of the above genes/proteins that are involved in axon transport are also involved in the transport of cellular organelles, such as mitochondria, endosomes/autophagosomes, and various vesicles (e.g., lysosomes, transport vesicles, and secretory vesicles).

*MAPT* binds microtubules and regulates the accessibility of tracks that kinesins and dynein use for trafficking. High tau occupancy inhibits kinesin-1 by steric hindrance, slowing anterograde movement of mitochondria, synaptic vesicle precursors, autophagosomes, endolysosomal vesicles. Microtubule stabilization by tau promotes efficient long-range axonal transport. Tau phosphorylation decreases microtubule binding leading to microtubule destabilization and transport failure. Misfolded tau aggregates physically obstruct vesicle movement.

*LRRK2* phosphorylates Rab GTPases (Rab8, Rab10, Rab12, Rab35, etc.), altering vesicle budding, docking, and movement. It binds microtubules and forms filamentous lattices that block kinesin and dynein. It regulates autophagosome-to-lysosome trafficking and endolysosomal maturation.

*PLEKHM1* (Pleckstrin Homology and RUN Domain Containing M1) interacts with microtubules, primarily by lysosome trafficking and fusion (Tabata et al., 2010; Mcewan et al., 2015). It is required for late stages of endolysosomal maturation, facilitating both endocytosis-mediated degradation of growth factor receptors and autophagosome clearance and promotes cargo traffic to lysosomes (Marwaha et al., 2017). Perturbations in this system could help to explain the accumulation of waste products in neurons. *PLEKHM1* is a key integrator of late endosomal, lysosomal, and autophagosomal trafficking as it binds Rab7, the major GTPase for late endosome and autophagosome transport, links autophagosomes to dynein–dynactin for retrograde transport along microtubules, and serves as a tether between autophagosomes and lysosomes via its HOPS-complex–interacting domain.

*STX6* (Syntaxin-6) is involved in targeting endosomal vesicles to the trans-Golgi network (TGN) and from the TGN to endosomal vesicles. It directs movement of vesicles from endosomes to the cell membrane. In addition, syntaxin-6 interacts with tau and enables its release from cells via secretory pathways. Hypomethylation in its promoter region contributes to *STX6* dysregulation in frontotemporal lobar degeneration and particularly in PSP (Rambarack et al., 2025). Syntaxin-6 also delays formation of prion fibrils. Furthermore, *STX6* is a risk gene in the neurodegenerative disease Creutzfeld-Jakob. Although misfolded prion proteins are the major cause of this disease misfolded protein tau occurs in some cases (Jones et al., 2024).

CNTN2 and NFASC play roles in forming adhesion complexes at nodes of Ranvier and the axon initial segment (AIS). Their cytoplasmic partners (ankyrin-G, spectrins) link to microtubules, creating barriers and filters that direct vesicle sorting.

*MOBP* (Myelin-Associated Oligodendrocyte Basic Protein) is synthesized by oligodendrocytes. It interacts with the microtubular network in oligodendrocytes, affecting microtubule organization and stability, which is crucial for myelin sheath formation in the central nervous system (Torii et al., 2022). *MOBP* plays a role as it is essential for myelin compaction; loss leads to axonal swelling, where vesicles accumulate due to impaired long-range transport.

*TRIM11* (Tripartite Motif Containing protein 11) has an E3 ubiquitin ligase activity. TRIMs can contribute to effectively remove the cellular misfolded proteins or protein aggregates via the unfolded protein system (UPS) pathway (Zhang et al., 2020). By mediating degradation of tau it protects against tauopathies. It also prevents misfolding of tau and dissolves tau fibrils. In AD *TRIM11* activity is reduced (Zhang et al., 2023). Owing to their association with tau, TRIM11 activity/function affects microfibrils.

*EIF2AK3* (Eukaryotic Translation Initiation Factor 2 Alpha Kinase 3) or PERK (Protein kinase RNA (PKR)-like ER Kinase) mediates reciprocal crosstalk between non-centrosomal microtubules and the endoplasmic reticulum (ER) (Sánchez-Álvarez et al., 2025). It also protects against tau aggregation suggesting a role in maintaining microtubule-associated protein stability under stress conditions (Ma et al., 2002) particularly those which involve the unfolded protein response (Smedley et al., 2021).

## 5 Possible differences between motor and CNS neurons

Proteins affecting microtubule-based trafficking and proteostasis (MAPT, LRRK2, PLEKHM1, STX6, TRIM11, EIF2AK3, CNTN2/NFASC) have disproportionately larger functional impact in motor neurons, while other (i.e., shorter CNS) neurons show more uniform effects across neuron types. Motor neurons (MNs) have exceptional dependence on long-range microtubule (MT) transport, polarized axons (up to >1 m in humans), and high proteostasis/autophagy demand. As a result, genes that regulate MT stability, axonal trafficking, endolysosomal fusion, and stress responses often show stronger functional consequences in MNs than in shorter or locally connected neurons (e.g., cortical interneurons) (De Vos et al., 2008). Brown (2003) (Millecamps and Julien, 2013; Maday and Holzbaur, 2016; Nixon, 2013; Ferguson, 2018; Sleigh et al., 2019) Table 2 shows these genes/proteins, their mechanistic explanations, and references.

**TABLE 2:** Genes/proteins that may have major impacts on long vs. short neurons that could disproportionately affect motor neurons.

Gene/Protein	Microtubule-related pathway	Mechanistic summary	Mechanistic explanation	Reference
CNTN2/NFASC	Axon initial segment and node scaffolding	Node of Ranvier and AIS organization depend on cytoskeletal scaffolds linked to MT bundles	Nodes of Ranvier organization critical for saltatory conduction along long MT-supported axons	Zonta et al. (2011), Susuki and Rasband (2008)
EIF2AK3 (PERK)	ER stress and proteostasis regulation	PERK modulates protein synthesis affecting axonal maintenance and MT-associated proteins	UPR activation alters tubulin/MAP synthesis and axonal protein supply; chronic PERK activation linked to MN degeneration	Ma et al. (2002) Smedley et al. (2021)
LRRK2	Rab phosphorylation and vesicle trafficking on MTs	LRRK2 regulates Rab GTPases controlling vesicle/lysosome transport along microtubules	Rab–kinesin/dynein coupling defects impair long-distance cargo delivery more severely	Steger et al. (2016), Rocha et al. (2020)
MAPT	Direct microtubule stabilization and spacing	Tau binds and stabilizes axonal microtubules; regulates kinesin/dynein transport over long distances	Long axons need dense tau-coated MT bundles; tau imbalance leads to axonal transport failure and distal degeneration first	Weingarten et al. (1975), Dixit et al. (2008)
PLEKHM1	Autophagosome–lysosome fusion on MT tracks	Rab7-dependent autophagosome fusion requiring MT-based trafficking	Distal autophagosomes must travel long distances; fusion defects cause axonal “traffic jams”	Mcewan et al. (2015)
STX6	Endosome–Golgi vesicle fusion	SNARE-mediated vesicle trafficking along microtubule routes	Retrograde/anterograde vesicle fusion steps depend on MT motors; MNs highly dependent on this flow	Bock et al. (1997)
TRIM11	Ubiquitin-mediated clearance of misfolded proteins	Prevents aggregate accumulation that disrupts MT-based transport	Proteostasis burden high due to axon length; aggregates impede MT tracks and motors	Zhang et al. (2023)

## 6 Indirect pathways

*SLCO1A2* (Solute Carrier Organic Anion Transporter Family Member 1A2) encodes the organic anion-transporting polypeptide 1A2 (OATP1A2) [3]. OATP1A2 is a membrane protein involved in the uptake of various substances such as organic anions across the cell membrane. In the brain *SLCO1A2* is mainly expressed in oligodendrocytes (Uhlén et al., 2015). Although there is presently no experimental evidence of a link between microtubules and OATP1A2, microtubules play a major role in intracellular trafficking of membrane proteins. Therefore, it is conceivable that microtubule disruption alters OATP1A2 location and function. The role of microtubules in intracellular trafficking further supports a potential role of microtubules in OATP1A2 function.

*C4A* encodes Complement Component 4A (CO4A) protein. It is part of the innate immune system. Furthermore, it is involved in synaptic pruning mainly in infancy up to puberty, but, to a much lesser extent throughout adult life (Westacott and Wilkinson, 2022). A role of C4A in the development of schizophrenia has been shown and attributed to complement-mediated immune processes (Gracias et al., 2022). Based on immunochemical and transcriptomic analyses this gene is a likely risk gene for PSP (Farrell et al., 2024). An interaction of C4A with microtubules has not been documented.

**FIGURE 1:**
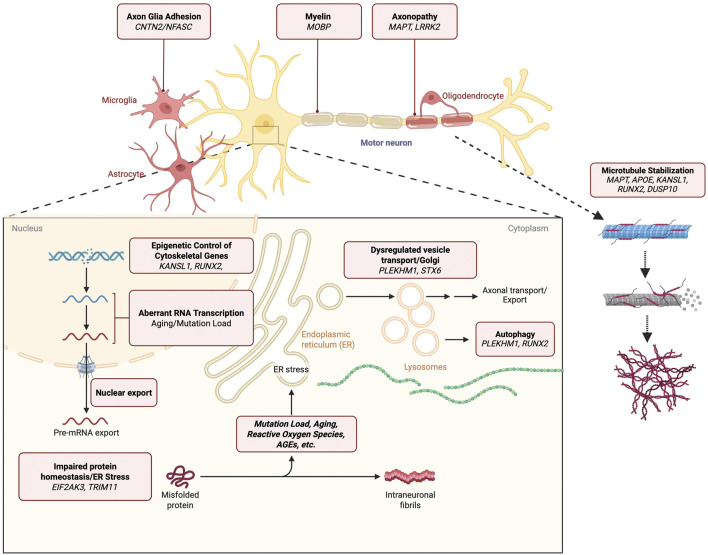
This schematic summarizes genes implicated in Progressive Supranuclear Palsy (PSP) that directly regulate microtubule stability and long-range axonal transport. *MAPT* stabilizes microtubule tracks required for kinesin- and dynein-mediated transport. *LRRK2*, through kinase-mediated modulation of Rab GTPases and microtubule binding, affects cargo trafficking, organelle positioning, and autophagosome motility. *CNTN2/NFASC* anchor cytoskeletal scaffolds at nodes of Ranvier, maintaining microtubule organization and vesicle-sorting barriers. *MOBP* contributes to myelin integrity, supporting axonal transport by maintaining axon caliber. *APOE*, *KANSL1*, *RUNX2*, and *DUSP10* modulate microtubule stability via lipid signaling, minus-end stabilization, acetylation control, and MAPK-mediated regulation, respectively. Together, these genes maintain polarized microtubule arrays essential for neuronal cargo movement.

**FIGURE 2:**
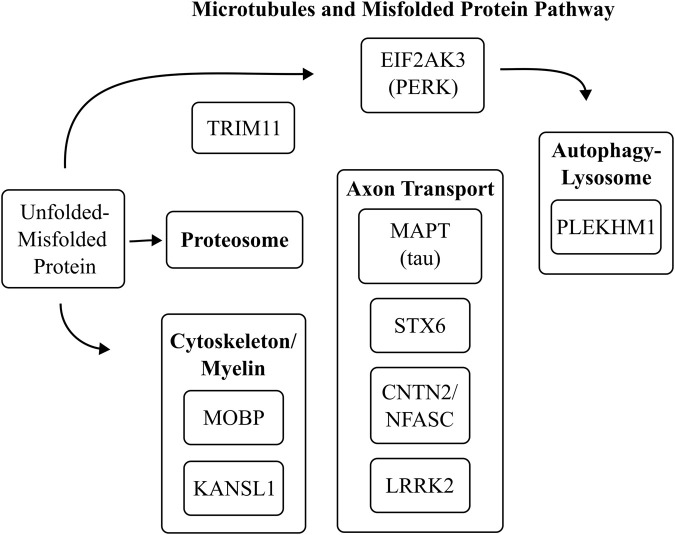
This diagram integrates genes that maintain cytoskeletal architecture, vesicle sorting, autophagy-lysosomal function, and protein quality control. *MAPT* regulates microtubule accessibility for transport motors. *PLEKHM1* links Rab7-positive autophagosomes to dynein and the HOPS complex, enabling autophagosome–lysosome fusion. *STX6* mediates endosome–Golgi trafficking and participates in tau secretion. *APOE* influences endosomal recycling and lysosomal degradation. *EIF2AK3 (PERK)* mediates the unfolded protein response (UPR), limiting ER stress and reducing misfolded protein accumulation. *TRIM11*, an E3 ligase, promotes clearance of misfolded tau through ubiquitin-proteasomal pathways. *MOBP*, *CNTN2/NFASC*, and *KANSL1* support cytoskeleton integrity, affecting vesicle trafficking. *SLCO1A2* and *C4A* play peripheral modulatory roles in membrane transport and complement signaling.

Shown in Figure 1 is the physiological function of the above genes. The encoded proteins are required for maintenance of structures that are involved in transporting nutrients, removal of waste, and the guidance of organelles essential for maintaining health and optimal functionality. These processes comprise the identification of mis-folded proteins, movement of secretory vesicles containing these misfolded proteins to the lysosome for degradation and export, and the homeostasis of intracellular macromolecular assemblies. In Figure 2 is a diagram that integrates genes that maintain cytoskeletal architecture, vesicle sorting, autophagy-lysosomal function, and protein quality control.

## 7 Discussion

Association of microtubular structure/function with MAPT has long been established in PSP (Morris et al., 2002; Spillantini and Goedert, 2013; Ballatore et al., 2007; Morfini et al., 2009). Many pathological mechanisms underlie abnormal microtubular function when MAPT is compromised. These include detachment of tau from microtubules due to hyperphosphorylation of tau, axonal degeneration/failure as an early sign of neurodegeneration, white matter disconnection, cytoskeletal collapse, and loss and gain of tau function. The present article describes these mechanisms for the majority of (13/15) PSP risk genes. The PSP risk gene-encoded proteins regulate intracellular transport, including nutrient delivery and clearance of aggregated tau and other proteins. Genetic variation alters functions related to axon integrity, organelle transport, cellular communication, and microtubule-guided waste disposal. Although direct evidence is lacking for *SLCO1A2* and *C4A*, theoretical considerations suggest that the *SLCO1A2*-encoded transporter OATP1A2 may influence microtubule homeostasis.

Disrupted microtubule regulation affects tau and/or the cytoskeleton. Altered tau expression or modification (e.g., hyperphosphorylation) promotes tau precipitation, impaired autophagy, and defective clearance, culminating in apoptosis. Alternatively, risk gene–mediated microtubule dysfunction may destabilize the cytoskeleton independently of tau. In both cases, impaired organelle trafficking leads to waste accumulation and neuronal death once damage becomes irreversible. These mechanisms align with PSP neuropathology, including neuronal loss, tufted astrocytes, neurofibrillary tangles, and coiled bodies, which are also characteristic of other tauopathies.

Microtubule alterations caused by PSP risk genes are likely subtle, and PSP, being multifactorial, may emerge only in combination with environmental stressors such as chronic oxidative stress, advanced glycation end products, and accumulation of misfolded proteins due to DNA damage–associated ER stress. The number and combination of inherited altered risk genes may further modulate disease severity and age of onset through cumulative effects of common polymorphisms. Risk gene–mediated microtubule dysfunction can impair: (a) tau function via altered MAPT RNA processing and hyperphosphorylation, destabilizing microtubules and cargo distribution; (b) directed transport of organelles, including mitochondria and vesicles, disrupting protein processing and degradation and promoting ER stress; and (c) extracellular export of degraded proteins through autophagy, leading to neuronal apoptosis.

The ER is central to protein folding and maturation, and its dysfunction results in misfolded protein accumulation and activation of the unfolded protein response (UPR). In neurodegenerative diseases, including tauopathies, UPR activation coincides with tau accumulation and promotes tau phosphorylation, a prerequisite for aggregation and neurofibrillary tangle formation (Hoozemans and Scheper, 2012). ER stress is induced by nutrient deprivation, oxidative stress, toxins, neonatal hypoxia-ischemia, and surgical stress (Guzel et al., 2017; Oakes and Papa, 2015; Carloni et al., 2014; Xin et al., 2022) and contributes to Parkinson’s disease, Alzheimer’s disease, amyotrophic lateral sclerosis, and multiple sclerosis (Grossmann et al., 2019; Colla, 2019; Santos and Ferreira, 2018; Lin et al., 2019; Halloran et al., 2020; Cunnea et al., 2011).

Microtubules are essential for autophagy, a catabolic process that removes damaged organelles and proteins to maintain cellular homeostasis (Eschbach and Dupuis, 2011; Glick et al., 2010). ER stress potently induces autophagy, and together these processes regulate cell survival (Aggarwal et al., 2024; Ogata et al., 2006). Dysregulated autophagy is implicated in multiple neurodegenerative diseases, and autophagy activation enhances clearance of amyloid beta in Alzheimer’s disease models (Liang and Sigrist, 2018; Paquet et al., 2018; Zhang et al., 2019).

Neuronal communication also occurs independently of synapses. Cytoskeletal microtubules support cognitive-like functions in single-cell organisms and are inhibited by anesthetics despite the absence of synaptic networks (Craddock et al., 2015). Anesthetics alter microtubule stability and act on cytoplasmic and membrane proteins, including tubulin and actin (Linganna et al., 2015; Perouansky, 2012; Franks and Lieb, 1984). Proteomic studies demonstrate anesthetic binding to numerous neuronal proteins involved in growth, division, and communication, all of which depend on microtubules (Xi et al., 2004; Pan et al., 2007; Pan et al., 2008). Although tubulin binding affinity is lower than for membrane proteins, its high abundance may render these interactions biologically significant.

## 8 Limitations of the current study

The genes listed in the current study were identified by genome-wide association studies (GWAS). Because of this, testing requires many variants (SNPs) and requires very stringent thresholds and may miss moderate and/or rare events. Effect sizes are often overestimated and the exact gene may not be accurate, due to linkage disequilibrium between the sentinal and functional variants. The latter point may be particularly relevant for the *CNTN2* and *NFASC* genes.
